# Retroperitoneal Angiomyolipoma: A Case Report and Review of the Literature

**DOI:** 10.1155/2013/457383

**Published:** 2013-11-05

**Authors:** Andrew Strahan, Jonathan King, Scott McClintock

**Affiliations:** Department of Urology, Gold Coast Hospital, Southport, QLD 4215, Australia

## Abstract

Retroperitoneal angiomyolipoma is a rare tumour that is difficult to diagnose preoperatively. We present a case of retroperitoneal angiomyolipoma that highlights its diagnostic dilemma. We also performed a literature review and present a review of retroperitoneal angiomyolipoma.

## 1. Introduction

Angiomyolipomas (AMLs) are composed of three tissue elements: mature adipose tissue, thick walled blood vessels, and smooth muscle cells. AMLs were originally classified as hamartomas but are now thought to belong to the family of perivascular epithelioid cell tumours (PEComas) [1]. AMLs are most commonly found in the kidney, and although most of them are sporadic, 20% are associated with tuberous sclerosis [2]. Rare cases of extrarenal AML have been reported affecting most commonly the liver and uterus, but to some extent the vagina, fallopian tubes, penis, lung, colon, nasal cavity, abdominal wall, and retroperitoneum [3]. Renal AMLs are present in 0.13% of the population [4] with only eleven retroperitoneal AMLs reported in the English literature [5]. Retroperitoneal AML patients present with abdominal pain, increasing abdominal girth, as incidental findings on imaging studies, or with spontaneous rupture [6]. Ultrasound and CT scan can correctly diagnose renal AML in 86% of cases [4]. Extrarenal AMLs, however, are more difficult to diagnose on imaging as they often lack fat densities [7]. Most cases of retroperitoneal AML have been treated with surgical excision, but successful conservative renal sparing management with angiographic embolisation has been reported in one case [8]. AMLs greater than 4 cm in size are more likely to be symptomatic and warrant intervention [9]. We present a case and review the literature to give an up-to-date perspective on retroperitoneal AML. 

## 2. Case Report

**Figure 1:**
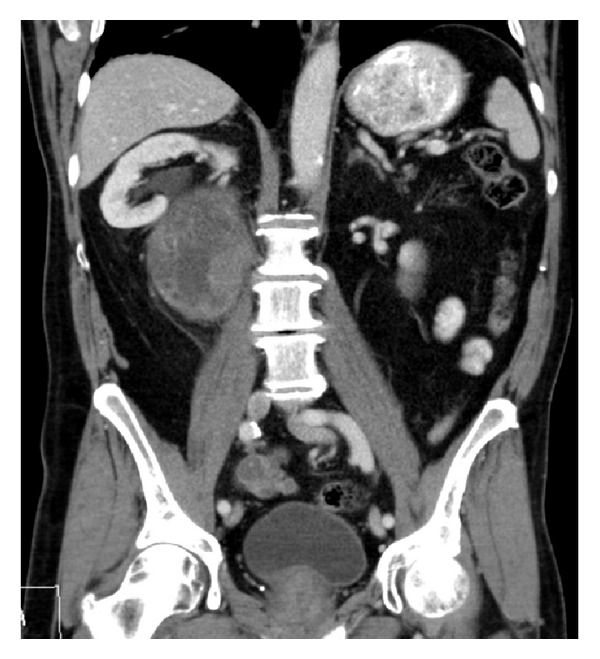
Right retroperitoneal mass displacing right kidney.

A 71-year-old man was referred from his general practitioner with hematochezia. He had no medical conditions; specifically, he did not have tuberous sclerosis but had a family history of a brother who died from colorectal cancer at the age of 60. Physical examination including digital rectal examination revealed a rectal mass. He had a significantly raised CEA (19.1 ug/L—RR < 5.0) and a slightly raised CA19-9 (35 U/mL—RR < 34) and went on to have a colonoscopy that showed a rectal mass with carcinoma confirmed on histological biopsy. Staging CT scan, as seen in Figure 1, showed thickening of the rectal wall but also revealed a large (9 × 9 × 10 cm) right sided retroperitoneal mass. The mass was heterogeneous with some calcification internally and peripherally with a smooth margin and surrounding soft tissue stranding. The mass displaced the right kidney superiorly and compressed the inferior vena cava. The patient was reviewed by the colorectal surgery and urology teams, and his case was discussed at the urology-radiology meeting. Radiologically, the lack of fat content of the retroperitoneal mass made it of concern for a metastatic lymph node. The patient went on to have an open ultralow anterior resection and resection of the retroperitoneal mass. The mass was not attached to the kidney but ureterolysis was required to dissect the right ureter that had been stretched laterally around the mass. The histology of the rectum revealed a T4N2 adenocarcinoma of the rectum. The histology of the retroperitoneal mass showed triphasic morphology comprising smooth muscle, thick walled blood vessels, and fat in a stellate network, consistent with angiomyolipoma (AML).

## 3. Discussion

Retroperitoneal neoplasms are uncommon and their preoperative diagnosis can be challenging. Causes of retroperitoneal tumours include lymphoma, liposarcoma, leiomyosarcoma, schwannoma, paraganglioma, neurofibromas, other rare tumours, and retroperitoneal lymph node metastasis, most commonly from testicular malignancies [10]. Many of these tumours have radiographically nonspecific features. Importantly, well-differentiated liposarcomas have smooth margins, a lobular contour, and predominate attenuation of fat, enhancing internal septations of soft tissue, which are the same radiographic findings of an angiomyolipoma [11].

Although AML is considered a benign tumour, a malignant epithelioid variant of AML has been described with 50% metastasising [12], with two cases in the literature having an extrarenal retroperitoneal primary [13, 14]. Immunohistochemical studies may be used to differentiate between classical AML and the epithelioid variant [12]. 

Our case highlights the difficulty in the diagnosis of retroperitoneal tumours and also reports on another case of retroperitoneal AML. 

Retroperitoneal AMLs are extremely rare tumours. Renal AMLs are easily identified on radiographic imaging but extrarenal AMLs are not as easily identified. Any retroperitoneal mass that is not convincing for classical AML could be a tumour with metastatic potential. A small asymptomatic retroperitoneal AML can be safely followed with surveillance, but larger lesions or atypical lesions should be surgically removed.
